# Research on Lower Limb Step Speed Recognition Method Based on Electromyography

**DOI:** 10.3390/mi14030546

**Published:** 2023-02-26

**Authors:** Peng Zhang, Pengcheng Wu, Wendong Wang

**Affiliations:** 1 Engineering Training Centre, Northwestern Polytechnical University, Xi’an 710000, China; 2 College of Automation, Northwestern Polytechnical University, Xi’an 710000, China; 3 College of Mechanical and Electrical Engineering, Northwestern Polytechnical University, Xi’an 710000, China

**Keywords:** electromyography (EMG), lower limbs, speed recognition

## Abstract

Wearable exoskeletons play an important role in people’s lives, such as helping stroke and amputation patients to carry out rehabilitation training and so on. How to make the exoskeleton accurately judge the human action intention is the basic requirement to ensure that it can complete the corresponding task. Traditional exoskeleton control signals include pressure values, joint angles and acceleration values, which can only reflect the current motion information of the human lower limbs and cannot be used to predict motion. The electromyography (EMG) signal always occurs before a certain movement; it can be used to predict the target’s gait speed and movement as the input signal. In this study, the generalization ability of a BP neural network and the timing property of a hidden Markov chain are used to properly fuse the two, and are finally used in the research of this paper. Experiments show that, using the same training samples, the recognition accuracy of the three-layer BP neural network is only 91%, while the recognition accuracy of the fusion discriminant model proposed in this paper can reach 95.1%. The results show that the fusion of BP neural network and hidden Markov chain has a strong solving ability for the task of wearable exoskeleton recognition of target step speed.

## 1. Introduction

The robot is a product of multidisciplinary intersection; since its birth, it has shown its unique advantages in every field, and gradually from the industry has expanded into military, medical, daily health care and other fields [1]; among them, exoskeleton robots have a broad range of application prospects in the medical health field, logistics and industrial manufacturing [2,3,4,5,6]. Particularly in the medical field, patients can complete a lot of physiological gait training with the help of the lower limb exoskeleton robot, in order to achieve the purpose of reestablishing the correct movement pattern as early as possible to participate in daily activities like healthy people [7].

The primary task for the exoskeleton to achieve the above functions is to accurately recognize the wearer’s movements. Song et al. [8] designed a classifier to identify five movement patterns including up the stairs, down the stairs, sit, stand and walk. Lopez-delis et al. [9] achieved knee motion pattern classification. Xi et al. [10] designed a classifier to recognize seven action patterns. Although a large number of studies have been able to perfectly realize the recognition and classification of different action patterns, this is not enough in real life. For example, a patient wants to walk slowly but the exoskeleton attached to them can only recognize when he is walking rather than squatting. Then it works at a speed that does not match the patient, which will have disastrous consequences. This paper will use running as an example to solve the problem of recognizing different speeds or magnitudes within the same movement mode.

The quality of the original information is directly related to the accuracy of human movement mode recognition and prediction. Andrea Bonci et al. [11] made a basic brain–computer interface (BCI) using EEG signals to research how targeted brain oscillation signals (or brainwaves) originate from a visual stimulus or a cognitive process and how they get acquired, processed and translated into commands. Peng et al. [12] used pressure as the original signal, then designed plantar pressure sensing shoes to collect the plantar pressure of the human body under different movement modes and extracted five characteristics. Zhang et al. [13] collected data under dynamic (walking) and static (sitting, standing and lying) activities of the elderly by using IMU. Gupta et al. [14] chose acceleration as the original signal; they used acceleration sensors tied to the waist to obtain them. However, there is an important problem with the above works. The information represented by pressure, acceleration and posture can only reflect the action information of the current exoskeleton wearer, but cannot directly predict the action information of the exoskeleton wearer.

In view of the above problem, this paper predicts human motion patterns based on EMG information. The EMG signal is a physiological signal sent by the activity of muscle neurons in the process of active human movements [15], which reflects neuromuscular activity to a certain extent [16]. EMG signals are forward-looking, independent, abundant and easy to operate [17,18]. Their prospectivity means that the occurrence of muscle action lags behind the generation of the corresponding EMG signal, which enables us to use the EMG signal as the input signal to control the exoskeleton and judge the user’s movement intention in advance [19]. Based on the above advantages, EMG signals are superior to other kinds of signals such as acceleration and pressure in human action pattern prediction.

Many algorithms have been successfully applied to human motion recognition based on EMG signals. Chen et al. [20] used multi-feature fusion with random forest (RF) to estimate ankle joint angle; Zhijun Li et al. [21] adapted a back propagation (BP) neural network to control strategy to assist humans to climb stairs. Tang et al. [22] adapted a BP neural network to learn the association between the sEMG signals and elbow angles under different loads. However, the above works concentrated on recognizing relatively discrete and discrepant motion. This paper’s main work is to study different speeds of the same action. For disabled people, the change of their walking speed is continuous and slow, but the above works do not fully exploit temporal information. This paper fully utilizes this characteristic, creatively coming up with the BP Neural Network–Hidden Markov hybrid model (BP-HMM), which improves the accuracy of step speed recognition.

## 2. Data Acquisition and Processing of Dual-Conduction Muscle Electrical Module

**Figure 1:**
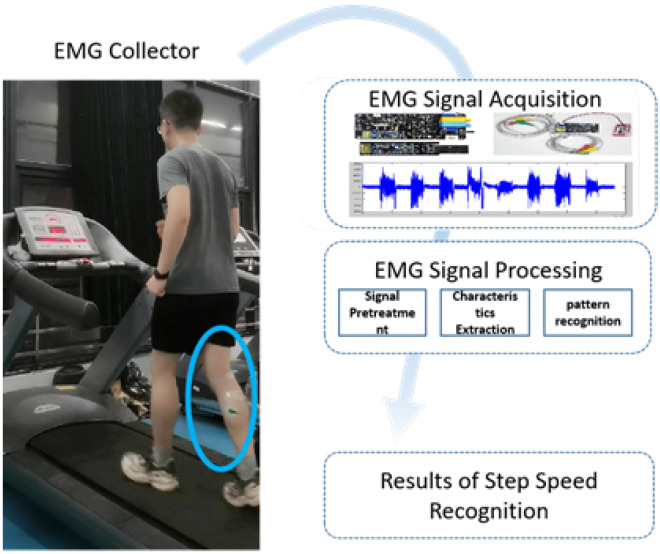
Basic flow chart of step pattern recognition.

As shown in Figure 1, the EMG signal has a relatively mature processing flow in the field of pattern recognition. Firstly, after the contact EMG signal sensor is attached to the selected muscles of the experimenter, the original signals are collected at the same time when the experimenter run on the treadmill at a specific speed. Secondly, the original signal is pre-processed including filtering and segmentation. Then, the features of the processed signals are extracted to obtain the training samples. Finally, the training samples are used to train the built network model offline to obtain the pattern recognition model.

### 2.1. Experimental Facilities

**Figure 2:**
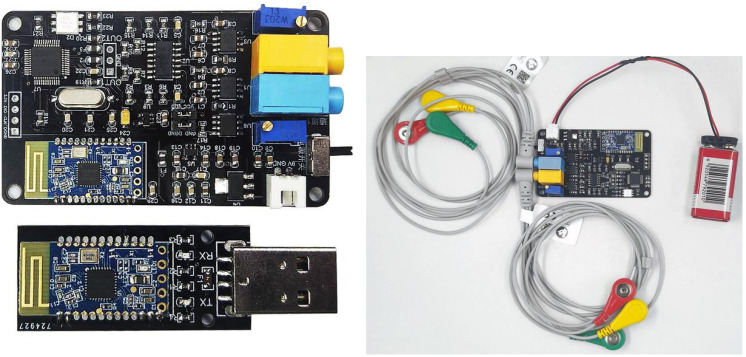
The physical diagram of the dual-conductance muscle electrical module.

In this study, the EMG signal acquisition of lower limb muscles is realized by the dual-conductance muscle electrical module, as shown in Figure 2. This module includes front-end analog circuit acquisition and back-end digital signal filtering processing. The front-end acquisition circuit collects the muscle electrical signals of the human arm or leg through two channels. After a series of signal amplification and filtering, the analog acquisition signal is output by the output port. The waveform of the muscle electrical signal can be observed directly through the filter. The back end uses a single chip microcomputer for digital filtering and collects the value of muscle electricity for processing to obtain power. Then it is sent to the host computer through Bluetooth 4.0. In the data collection, the testers are required to run at the speeds of 3, 4, 5, 6, 7, 8 and 9 km/h on the treadmill in this experiment. Compared with the complexity of the open-air environment, the indoor treadmill can effectively ensure that the tester can complete the data acquisition at a stable speed and change the speed of the treadmill instantaneously, so that the tester can change the running speed in a short time. At this time, the signal collected by the sensor is the signal with the tester’s speed changing. All the above signals will be stored as original data.

### 2.2. Data Collection and Preprocessing

According to the related research of human muscle movement theory, the motion state of human lower limbs is mainly a comprehensive result of the coordinated control of lower limb muscles, and the interaction between the active muscles and the antagonistic muscles to maintain the dynamic balance of the human body during exercise.

According to Qin Geyu et al. [23], knee flexion in the lower limb joint model is mainly composed of the following groups of muscles, including the vastus medialis muscle and vastus lateralis muscle, semitendinosus and biceps femoris muscle, et al. In addition to the above muscle clusters related to the knee joint, the ankle joint also plays an important role in the movement of lower limbs, including the selection of medial gastrocnemius head, lateral gastrocnemius head and soleus, et al. in the metatarsal flexor group of the ankle joint. In this paper, the above part of the muscle groups involved in the lower limbs of the human body during running are selected. According to the data and experimental results, two groups of muscle groups including the extrafemoral rectus muscle and vastus medialis that have a greater impact on the walking speed in the above muscle groups are selected as the experimental objectives. Based on this, the relevant data are extracted for processing and analysis to reduce the redundant signals that are weakly related to the walking speed, which improves the accuracy and computational efficiency of the algorithm.

**Figure 3:**
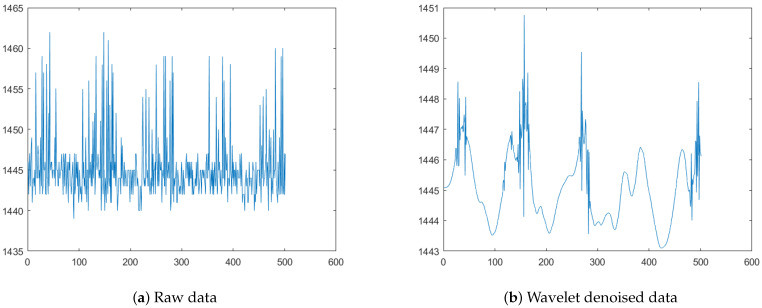
EMG signals before and after noise reduction. (The left image is the original signal and the right image is the signal after db5 wavelet filtering).

Due to the complexity of human muscle structure and the accuracy of the acquisition equipment itself, the EMG signal is vulnerable to noise in the acquisition process. Noise can be divided into high frequency noise and low frequency noise according to its own frequency band. Low frequency noise includes electrical signals generated by human tissues outside the target muscle group, and high frequency noise includes electromagnetic interference in the experimental site and noise of the equipment itself. The dual-conduction muscle electrical module used in this experiment has a certain hardware filtering function but, in order to ensure the accuracy of the data, this experiment uses algorithm filtering to carry out the secondary processing of the original data. In this paper, the wavelet denoising method is used and wavelet dp5 is used as the fundamental wave to realize signal denoising. Taking the rectus femoris muscle during walking as an example, the signals before and after noise reduction are shown in Figure 3.

For the data signal flow generated by the dual-conduction muscle electrical module, this paper adopts the data segmentation method based on time window to extract the phased effective numbers in the data flow. The time window plays an important role in the processing of the EMG signal pattern. Many studies [24,25,26] have discussed this problem in depth. Based on the above research, in gait recognition, we need to detect the initial moment of gait and, on this basis, conduct window division. During the gait cycle division, according to the research conclusion of Hao Jinghan’s gait cycle division based on a TK energy operator algorithm and real-time monitoring results during data collection [27], it is found that the gait cycle control between 1 and 1.3 s is the most accurate. Based on the above analysis, the EMG signal was processed by sliding window; the window is set at 200 ms (including 200 sampling points). As shown in Figure 3b, similar time domain and frequency domain characteristics are displayed between the 100th to 200th and the 200th to 300th sampling points, etc. Combined with the selection of the length of sliding window in [24,25,26], we choose 200 ms as the length of time window and the overlapping ratio is set at 50%, which can not only ensure the efficiency of data use but also does not cause data loss. In the process of data acquisition, the current sampling time is taken as the time node and the data of the first 1 s are intercepted as the sliding time window.

EMG signal feature extraction methods are generally divided into time domain, frequency domain and time–frequency domain. See Table 1 for some eigenvalues.

In this paper, we use principal component analysis, selecting 10 characteristics such as root mean square, corrected average absolute value, zero crossing times and maximum and minimum values for observation in the time domain. In the frequency domain, the spectrum of the EMG signal can intuitively reflect the difference between different speeds. Therefore, in this paper, four eigenvalues such as median frequency and mean frequency are extracted from the spectrum of EMG signal as the observation eigenvalues in the frequency domain. Since the dual-conduction EMG module can collect the EMG signals of two muscle groups at the same time, combined with the characteristics of EMG signals of different muscle groups, the rectus femoris and extrafemoral muscles are selected as the signal sources in this experiment. Finally, the eigenvalue matrix of 2 × 14 is used as the input sample of the model in this paper.

## 3. BP-HMM

The BP-HMM model consists of the BP neural network model and HMM. The BP neural network is based on the error back propagation algorithm, which belongs to the forward network to solve the nonlinear separability problem. The BP network is the core part of the forward neural network and the essence of the artificial neural network. It is widely used in pattern recognition, approximation, regression and other fields. HMM is a statistical model. It is achieved by the following steps. Firstly, a hidden Markov chain generates a state random sequence. Then each state in the state random sequence generates the corresponding observation. Because the hidden state observation probability matrix of HMM cannot be directly obtained, this paper firstly uses the BP neural network model to identify the sample offline and obtains the observation probability matrix required by the HMM model. Then, according to the observation value, the hidden state probability is obtained by the Viterbi algorithm and the step speed is identified online. The accuracy of step speed recognition can be improved by using this model.

### 3.1. BP Neural Network Model

**Figure 4:**
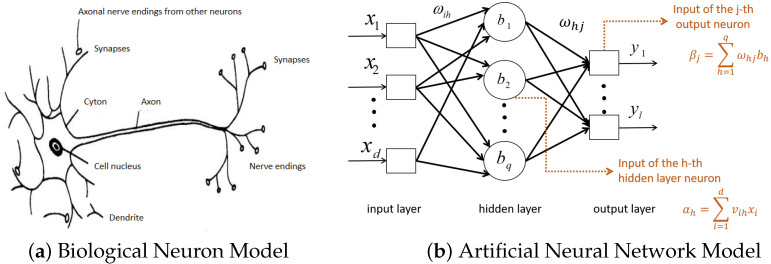
Biological and mathematical models of neural networks.

The artificial neural network (Figure 4b) is a data processing model that simulates biological neurons (Figure 4a). It is calculated by a large number of artificial neurons connected with each other and changes its structure according to the external information. The operation process is mainly to adjust the weights between neurons to model the input data and ultimately has the ability to solve practical mathematical problems.

The BP algorithm is a supervised learning algorithm. Its purpose is to adjust the network parameters by using the mean square error between the actual output and the expected output of the network based on the gradient descent strategy, so that the error between the expected output and the actual output is minimized. The input $x_{1},x_{2},\ldots ,x_{N}$ are *N* eigenvalues extracted from the EMG signals in the previous section and the output $y_{1},y_{2},\ldots ,y_{M}$ are *M* different step speeds to be identified. The network model structure is shown in Figure 4b.

#### 3.1.1. Definition of Variables

In Figure 4b, the number of input neurons is d=28, the number of hidden neurons is q=10 and the number of output neurons is l=7. The *i*-th neuron in the input layer is denoted as $x_{i}$, the *h*-th neuron in the hidden layer is denoted as $b_{h}$ and the *j*-th neuron in the output layer is denoted as $y_{j}$. The connection weight from $x_{i}$ to $b_{h}$ is $\omega_{ih}$ and from $b_{h}$ to $y_{j}$ is $\omega_{hj}$. Both the transfer functions of the hidden layer and the hidden layer use the sigmoid function.

#### 3.1.2. Formula Derivation

Strict mathematical proof of the formula can be seen in Reference [39] and this paper only makes a simple explanation.

For training sample $\left(x_{k},y_{k}\right)$, suppose the output ${\hat{y}}_{k}=\left({\hat{y}}_{1}^{k},{\hat{y}}_{2}^{k},\ldots ,{\hat{y}}_{M}^{k}\right)$ of BP network:

(1) $${\hat{y}}_{j}^{k}=f\left(\beta_{j}-\theta_{j}\right),j=1,\ldots ,M,$$

Define the deviation of the training sample in the network by mean square error:

(2) $$E_{k}=\frac{1}{2}\sum_{j=1}^{M}{\left({\hat{y}}_{j}^{k}-y_{j}^{k}\right)}^{2},$$

In each iteration, the generalized perceptron learning rule is used to update the parameters. For any parameter *u*, the updating rule is:

(3) u+Δu→u,

Based on the gradient descent strategy, the parameters are adjusted in the negative gradient direction of the target. For the error $E_{k}$ given by Formula (2), the learning rate is set to be η:

(4) $$\Delta \omega_{hj}=-\eta \frac{\partial E_{k}}{\partial \omega_{hj}}=-\eta \frac{\partial E_{k}}{\partial {\hat{y}}_{j}^{k}}\cdot \frac{\partial {\hat{y}}_{j}^{k}}{\partial \beta_{j}}\cdot \frac{\partial \beta_{j}}{\partial \omega_{hj}},$$

By definition $\beta_{j}$:

(5) $$b_{h}=\frac{\partial \beta_{j}}{\partial \omega_{hj}},$$

By properties of sigmoid function:

(6) $$g_{j}=-\frac{\partial E_{k}}{\partial {\hat{y}}_{j}^{k}}\cdot \frac{\partial {\hat{y}}_{j}^{k}}{\partial \beta_{j}}={\hat{y}}_{j}^{k}\left(1-{\hat{y}}_{j}^{k}\right)\left(y_{j}^{k}-{\hat{y}}_{j}^{k}\right),$$

By substituting Formula (5) and (6) into Formula (4), the learning formula in the BP algorithm can be obtained:

(7) $$\Delta \omega_{hj}=\eta g_{j}b_{h},$$

Similarly, the following parameters can be obtained to obtain the learning formula:

(8) $$\Delta \theta_{j}=-\eta g_{j},$$

(9) $$\Delta v_{ih}=\eta e_{h}x_{i},$$

(10) $$\Delta \gamma_{h}=-\eta e_{h},$$

Among them:

(11) $$e_{h}=b_{h}\left(1-b_{h}\right)\sum_{j=1}^{l}\omega_{hj}g_{j},$$

#### 3.1.3. Algorithmic Process

**Figure 5:**
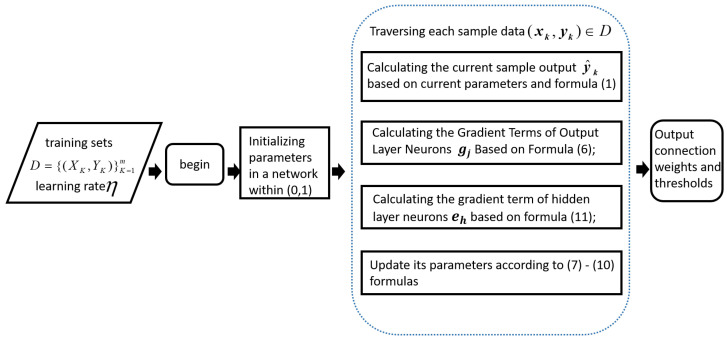
BP algorithm process.

On the basis of the iterative calculation formula of the above parameters, the algorithm flow of the classical BP neural network can be obtained as shown in Figure 5.

### 3.2. HMM

#### 3.2.1. External Representation of HMM

HMM is a classical statistical model of machine learning, which has been widely used in the fields of language recognition, natural language processing and pattern recognition. With the invention and rapid development of sensor technology, this model has also been used in the field of human motion intention recognition. The hidden Markov model uses the following representation [40]:

(12) λ={Q,V,π,A,B},

where *Q* is the set of all possible hidden states, *V* is the set of all possible observed states, π is the initial probability matrix, *A* is a state transition matrix and *B* is the observation probability matrix, namely:

(13) $$Q=\left\{q_{1},\ldots ,q_{N}\right\},V=\left\{v_{1},\ldots ,v_{M}\right\},$$

In this paper, the characteristic values extracted from the EMG signal are the observation states and the step speeds are the hidden states. Therefore, N=28, $\left\{q_{1},\ldots ,q_{28}\right\}$ corresponds to 28 eigenvalues; M=7, $\left\{v_{1},\ldots ,v_{7}\right\}$ represents, respectively, 3, 4, 5, 6, 7, 8, 9 km/h; π represents the initial probability matrix of the hidden state, namely the probability of each step being selected in the first step. Due to the randomness of the experiment, this paper selects uniform distribution as the initial probability matrix.

(14) $$\pi =[\underset{M}{\underset{︸}{\frac{1}{M},\ldots ,\frac{1}{M}]}},$$

**Figure 6:**
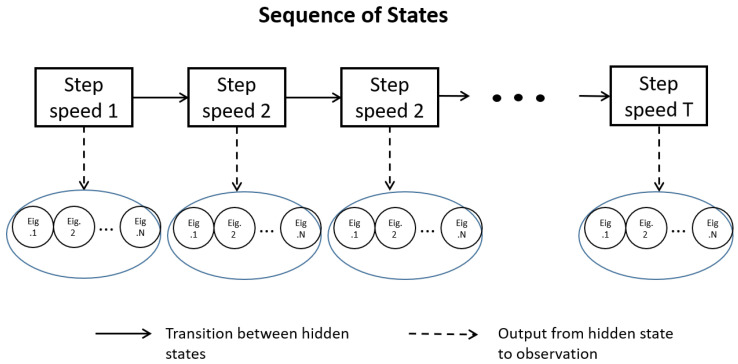
State sequence and observation sequence in step speed detection.

After a period of time *T*, the state sequence of length *T* is generated: $I=\{i_{1},i_{2},i_{3}\ldots ,i_{T}\}$ and the corresponding observation sequence: $O=\{o_{1},o_{2},o_{3}\ldots ,o_{T}\}$; Figure 6 shows the relationship between the hidden state and the observed state with time.

#### 3.2.2. Intrinsic Factors of HMM

The above mainly discusses the external representation of HMM and the following mainly discusses its internal factors. Before discussing the intrinsic factors of the hidden Markov model, two properties are listed as follows:

**Homogeneous Markov property:** the hidden state of the hidden state Markov chain at any time only depends on the hidden state of the previous time and has nothing to do with the earlier hidden state; this, of course, has nothing to do with the observation. This property can be expressed by conditional probability as follows:

(15) $$P\left(i_{t}|i_{t-1},o_{t-1},i_{t-2,}o_{t-1},\ldots ,i_{1},o_{1}\right)=P\left(i_{t}|i_{t-1}\right),$$

**Observation independence:** the observation at any time is only related to the hidden state at that time; this property can be expressed by conditional probability as follows:

(16) $$P\left(o_{t}|i_{t},i_{t-1},o_{t-1},i_{t-2,}o_{t-1},\ldots ,i_{1},o_{1}\right)=P\left(o_{t}|i_{t}\right),$$

*A* is a state transition matrix; its essence is a Markov chain transition probability matrix; all potential hidden state numbers are *N*, so *A* is an N×N matrix:

(17) $$a_{ij}=P\left(i_{t+1}=q_{j}|i_{t}=q_{i}\right),$$

where i=1,2,…,N, j=1,2,…,N. Obviously, $a_{ij}$ represents the probability of hidden state *i* transferring to hidden state *j*. Through the analysis of the real-time signal in the field experiment and combined with the actual law of walking transformation, this paper argues that the walking transformation is continuous, and has a strong correlation between $q_{t+1}$ and $q_{t}$, which conforms to the nature (1). The Gaussian distribution is used as the transfer probability to simulate the probability of walking speed conversion, which is closer to the reality. The specific calculation is as follows:

(18) $$A_{i}∼N\left(i,1\right),1\leq i\leq 7,i\in Z,$$

$A_{i}$ is the *i*-th row of *A*, which indicates the probability that the current pace moves to the next pace, and obeys the normal distribution with mean and variance 1, where $A_{ii}$ is the current speed state and $A_{ii}$ is also the mean value of the normal distribution, so the maximum probability of the next step after each step conversion remains the current state.

*B* is the observation probability distribution matrix, also called the confusion matrix, representing the probability when the hidden state is $q_{i}$ and the output is the observation state $v_{j}$.

(19) $$b_{ij}=P(o_{t}=v_{j}\left|i_{t}=q_{i}\right),$$

where i=1,2,…,N, j=1,2,…,N. Obviously $b_{ij}$ refers to the probability that the corresponding generated observation state is $v_{j}$ when time is *t* and hidden state is $q_{i}$. $b_{ij}$ is established by the BP model. The offline BP recognition method is used to calculate the probability that the BP model recognizes the observed $v_{j}$ when the current hidden state is $q_{i}$.

After obtaining HMM, λ={Q,V,π,A,B} from experimental data, the hidden state sequence is decoded by the Viterbi algorithm (forward–backward algorithm). The specific process of the Viterbi algorithm is as follows:

**Initialize local state**

(20) $$\delta_{1}\left(i\right)=\pi_{i}{b_{i}}_{o_{1}},i=1,2,\ldots ,N,$$

(21) $$\psi_{1}\left(i\right)=0,i=1,2,\ldots ,N,$$

**Dynamic recursive:** recursive local state t=1,2,…,T for dynamic programming:

(22) $$\delta_{t}\left(i\right)=\max_{1\leq j\leq N}\left[\delta_{t-1}\left(j\right)a_{ji}\right]{b_{i}}_{o_{t}},i=1,2,\ldots ,N,$$

(23) $$\psi_{t}\left(i\right)=\arg\max_{1\leq j\leq N}\left[\delta_{t-1}\left(j\right)a_{ji}\right],i=1,2,\ldots ,N,$$

The probability of the maximum hidden state sequence is solved as follows: calculating the maximum $\delta_{T}\left(i\right)$ at the end time *T*, that is, the maximum probability of the hidden state sequence, and calculating the $\delta_{T}\left(i\right)$ under this condition, that is, the most likely hidden state at time *T*.

(24) $$P^{*}=\max_{1\leq j\leq N}\left[\delta_{T}\left(i\right)\right],$$

(25) $${i_{T}}^{*}=\arg\max_{1\leq j\leq N}\left[\delta_{T}\left(i\right)\right],$$

**Backdate:** For t=T−1,T−2,…,1:

(26) $${i_{t}}^{*}=\psi_{t+1}\left({i_{t+1}}^{*}\right),$$

Finally, the most possible hidden state sequence $I^{*}=\left\{{i_{1}}^{*},{i_{2}}^{*},\ldots ,{i_{T}}^{*}\right\}$ is the predicted step sequence.

## 4. Model Training and Experimental Results

**Figure 7:**
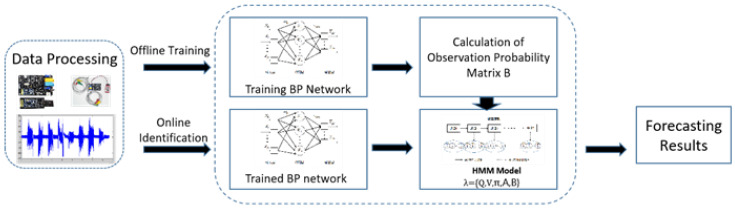
Flow chart of BP-HMM speed prediction model.

This paper combines the BP network and HMM model to get the BP-HMM model as a recognition model. Firstly, the BP neural network is trained offline according to the observed data and the confusion matrix of the recognition results is used as the observation probability matrix of the HMM model. Then the BP-HMM speed prediction model is obtained. In online prediction, the BP-HMM model is used for identification and the final specific process is shown in Figure 7.

**Figure 8:**
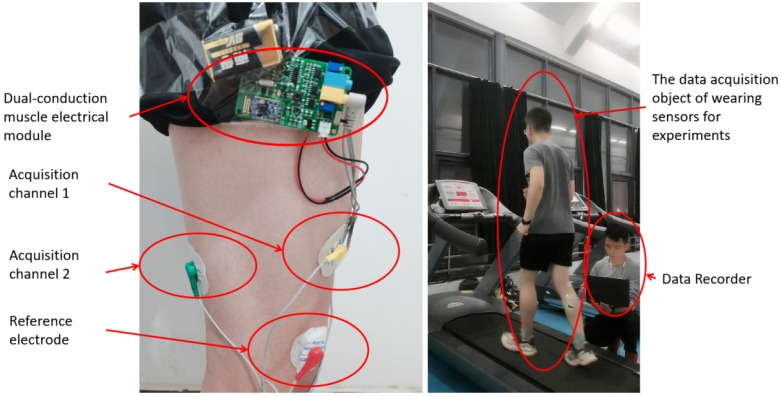
Experimental scene diagram.

**Figure 9:**
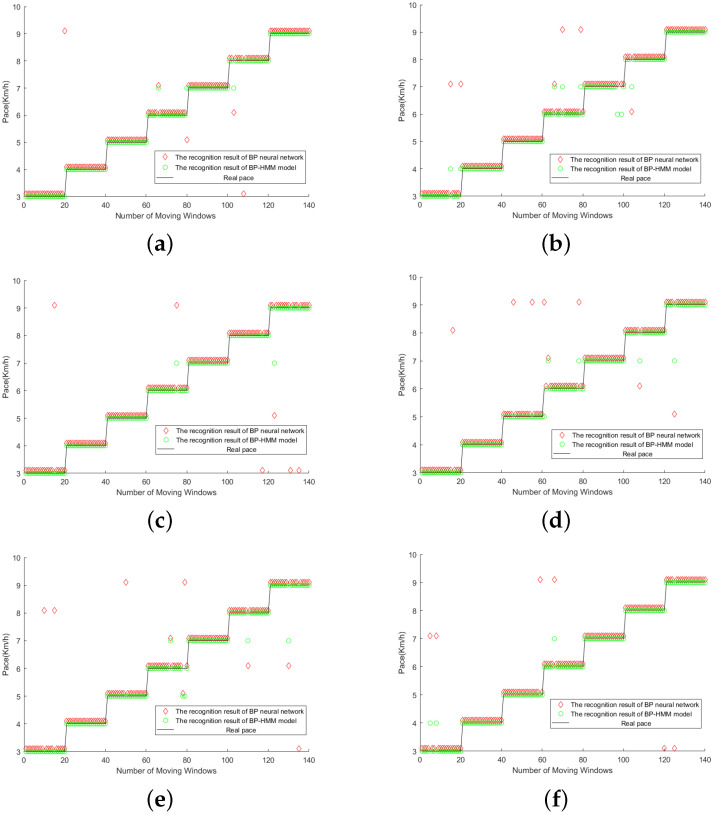
(**a**–**f**) Comparison of some test results (BP-HMM and BP neural network). In order to prove the robustness of the model, we marked some samples and stored them in the database, randomly extracted some data from them, identified them by different models, and obtained comparative results.

The experimenters walked at seven different speeds from 3 km/h to 9 km/h on the treadmill (Figure 8) and each speed extracted 300 to 400 windows of time. Finally, considering the large data error in the first and last sections of each speed, they were eliminated. Finally, the data of 300 windows in the middle section of each speed were retained as the test, namely, the size of the data set was 2100. To prove the robustness of the model, we selected three different experimenters, including two male members and one female member. Each experimenter repeated the above behaviors to obtain muscle electrical signals at asynchronous speeds and stored them in a database. Each database was used to train the model. Twenty groups of data were randomly selected from the data corresponding to asynchronous speeds in the database, a total of 140 groups of data, and multiple rounds of tests were conducted on the results. The test results are compared with the recognition results of the BP neural network. Part of the test results are shown in Figure 9.

Judging from the results in Figure 9, the recognition accuracy of BP-HMM is better than that of the neural network model and, since the data of each test is randomly extracted, it proves that BP-HMM has good robustness. No matter how complex the neural network is, it can be used as a part of BP-HMM, so BP-HMM always has better performance and discrimination ability than any neural network in the problem of human walking speed recognition.

In order to specify the results obtained from our model, we choose one set of results to illustrate in detail. When we randomly selected 140 sets of data from one of the experimenters’ databases, we classify them using two models, the BP neural network and the BP-HMM model. This set of identification results using the three-layer BP network are shown in Table 2.

It can be seen from Table 2 that there are many errors in the recognition results of the BP neural network, and the errors are irregularly distributed and difficult to correct. The recognition result is denoted as the observation probability matrix *B* of the BP-HMM model:

(27) $$B=\;\left[\begin{array}{ccccccc} 5.469\times 10^{-1} & 0 & 0 & 2.188\times 10^{-1} & 0 & 1.875\times 10^{-1} & 4.690\times 10^{-2}\\ 0 & 9.296\times 10^{-1} & 0 & 2.820\times 10^{-2} & 2.820\times 10^{-2} & 0 & 1.410\times 10^{-2}\\ 0 & 1.450\times 10^{-2} & 7.826\times 10^{-1} & 0 & 1.719\times 10^{-1} & 0 & 4.690\times 10^{-2}\\ 1.429\times 10^{-1} & 0 & 0 & 6.786\times 10^{-1} & 1.786\times 10^{-1} & 0 & 0\\ 6.380\times 10^{-2} & 0 & 1.064\times 10^{-1} & 1.277\times 10^{-1} & 5.532\times 10^{-1} & 0 & 1.489\times 10^{-1}\\ 8.820\times 10^{-2} & 0 & 0 & 0 & 0 & 9.118\times 10^{-1} & 0\\ 7.690\times 10^{-1} & 0 & 0 & 0 & 1.026\times 10^{-1} & 5.130\times 10^{-2} & 7.682\times 10^{-1} \end{array}\right]$$

The state transition matrix *A* can be obtained from Formula (18):

(28) $$\begin{array}{c} A= \end{array}\;\;\left[\begin{array}{ccccccc} 3.9\times 10^{-1} & 2.4\times 10^{-1} & 5.4\times 10^{-2} & 4.43\times 10^{-3} & 1.34\times 10^{-4} & 1.49\times 10^{-6} & 6.08\times 10^{-9}\\ 2.4\times 10^{-1} & 3.9\times 10^{-1} & 2.4\times 10^{-1} & 5.4\times 10^{-2} & 4.43\times 10^{-3} & 1.34\times 10^{-4} & 1.49\times 10^{-6}\\ 5.4\times 10^{-2} & 2.4\times 10^{-1} & 3.9\times 10^{-1} & 2.4\times 10^{-1} & 5.4\times 10^{-2} & 4.43\times 10^{-3} & 1.34\times 10^{-4}\\ 4.43\times 10^{-3} & 5.4\times 10^{-2} & 2.4\times 10^{-1} & 3.9\times 10^{-1} & 2.4\times 10^{-1} & 5.4\times 10^{-2} & 4.43\times 10^{-3}\\ 1.34\times 10^{-4} & 4.43\times 10^{-3} & 5.4\times 10^{-2} & 2.4\times 10^{-1} & 3.9\times 10^{-1} & 2.4\times 10^{-1} & 5.4\times 10^{-2}\\ 1.49\times 10^{-6} & 1.34\times 10^{-4} & 4.43\times 10^{-3} & 5.4\times 10^{-2} & 2.4\times 10^{-1} & 3.9\times 10^{-1} & 2.4\times 10^{-1}\\ 6.08\times 10^{-9} & 1.49\times 10^{-6} & 1.34\times 10^{-4} & 4.43\times 10^{-3} & 5.4\times 10^{-2} & 2.4\times 10^{-1} & 3.9\times 10^{-1} \end{array}\right]$$

**Figure 10:**
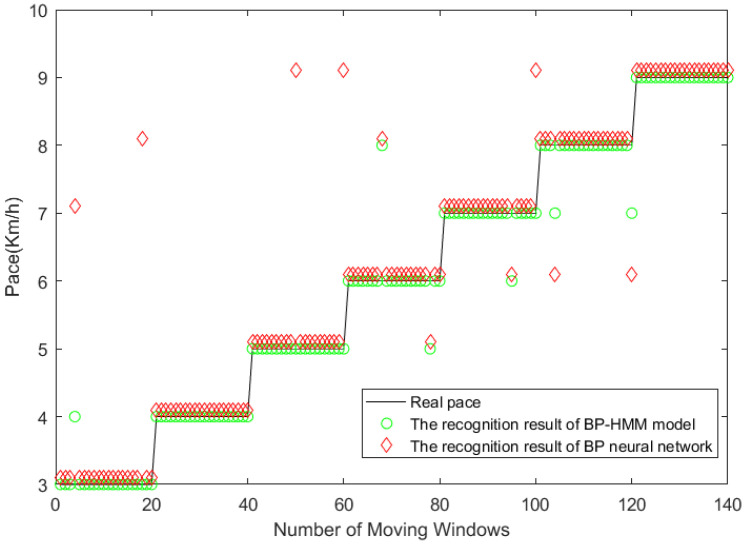
Comparison of recognition results between BP-HMM model and BP model under continuous walking.

The state transition matrix *A* and observation probability matrix *B* are substituted into Formula (12) to obtain the complete BP-HMM. The online recognition test of the model was carried out. The experimenter walked on the treadmill at seven continuous fixed speeds. A total of 140 sets of data were randomly collected from 20 windows for each speed. The three-layer BP neural network and the BP-HMM is used to identify the data set online. The identification results are shown in Table 3 and Table 4, respectively. Comparing the two identification results, the BP-HMM model proposed in this paper has the following advantages compared with the three-layer BP network:

(1) The overall recognition rate is improved on the basis of high performance of the BP network, from 92.86% to 95.00%.

(2) The absolute deviation and relative deviation of the BP network recognition results are greater than the absolute deviation and relative deviation of the BP-HMM model, that is, the error recognition results of the proposed model are only in the adjacent state of its real value. Under the premise that the recognition results are not very accurate, more than 90% of the deviation remains within the unit error of the correct pace. The false recognition results of the BP network are randomly distributed in different speeds and the error is large.

(3) Due to the physiological structure of the human body, under normal circumstances, the pace of the human body changes to a stable change, which means the large probability of the pace at a certain moment does not change at the next moment or changes to a certain pace of its adjacent state. Therefore, this paper incorporates this feature into the model and successfully solves the problem of the large identification error of the BP neural network at different step speed transition moments, as shown in Figure 10. At the time of pace change, the BP-HMM model does not misidentify the samples.

## 5. Conclusions

This paper presents a lower limb gait recognition method for human exoskeleton wearers based on the BP-HMM model. Firstly, according to the EMG characteristics of different muscle groups and the walking characteristics in pattern recognition, the BP-HMM recognition algorithm is proposed and used to divide the walking speed into seven states. Then, by extracting and analyzing the eigenvalues of EMG signals at different muscle groups of exoskeleton wearers, two muscle groups with obvious characteristics and large differences are selected as the final analysis objects. Then the original data are analyzed in time domain and frequency domain, and 28 characteristic values are extracted from each muscle group. A total of 28 eigenvalues of two muscle groups are used as the total feature set. Finally the BP-HMM model is used to identify the current pace online. The final results show that the overall speed recognition rate of the model reaches 95% and each index is better than the single BP neural network model. The traditional recognition algorithm does not take into account the general rules of human walking and this paper improves the HMM state matrix according to the general rules of human walking, which can more accurately describe the speed intention of exoskeleton wearers.

In this paper, the traditional recognition algorithm is improved in principle, which improves the recognition accuracy of human walking recognition in this specific field. At the same time, it avoids the huge difference between recognition state and walking intention, and basically solves the problem of mutation of recognition results and inaccurate recognition intention in this field. The foundation has been laid to ensure movement coordination between the exoskeleton and its wearers.

## Figures and Tables

**Table 1:** List of characteristic values of typical EMG signals.

EMG Eigenvalue Name	Feature Type	Reference
Root mean square, RMS	Time domain feature	[28,29,30]
Mean absolute value, MAV	Time domain feature	[31,32]
Slope sign change, SSC	Time domain feature	[33]
Waveform length, WL	Time domain feature	[34,35]
Zero crossing, ZC	Time domain feature	[34,35]
Median frequency, MDF	Frequency domain feature	[36,37]
Mean power, MNF	Frequency domain feature	[37,38]

**Table 2:** BP neural network model identifies confusion matrix offline.

Real Pace (km/h)	Identification Result (km/h)						
	3	4	5	6	7	8	9
3	0.5469	0	0	0.2188	0	0.1875	0.0469
4	0	0.9296	0	0.0282	0.0282	0	0.0141
5	0	0.0145	0.7826	0	0.1719	0	0.0469
6	0.1429	0	0	0.6786	0.1786	0	0
7	0.0638	0	0.1064	0.1277	0.5532	0	0.1489
8	0.0882	0	0	0	0	0.9118	0
9	0.0769	0	0	0	0.1026	0.0513	0.7682

**Table 3:** BP neural network model identifies confusion matrix online.

Real Pace (km/h)	Identification Result (km/h)						
	3	4	5	6	7	8	9
3	95%	0	0	0	5%	0	0
4	0	95%	0	0	0	5%	0
5	0	0	95%	0	0	5%	0
6	0	0	5%	85%	0	5%	5%
7	0	0	5%	10%	90%	0	0
8	0	0	0	5%	0	90%	5%
9	0	0	0	0	0	0	100%

**Table 4:** BP-HMM model identifies confusion matrix online.

Real Pace (km/h)	Identification Result (km/h)						
	3	4	5	6	7	8	9
3	95%	0	0	0	0	0	0
4	0	100%	0	0	0	0	0
5	0	0	100%	0	0	0	0
6	0	5%	90%	0	0	5%	0
7	0	0	0	5%	95%	0	0
8	0	0	0	0	5%	95%	0
9	0	0	0	0	0	0	100%

## Data Availability

The data presented in this study are available on request from the corresponding author.
